# Glucocorticoid receptor action in metabolic and neuronal function

**DOI:** 10.12688/f1000research.11375.1

**Published:** 2017-07-24

**Authors:** Michael J. Garabedian, Charles A. Harris, Freddy Jeanneteau

**Affiliations:** 1 Department of Microbiology, New York University School of Medicine, Alexandria Center for Life Sciences, 450 East 29th Street, Room 324, New York, NY, 10016, USA; 2 Department of Internal Medicine, Division of Endocrinology, Metabolism and Lipid Research, Washington University School of Medicine, St. Louis, MO, 63110, USA; 3 Departments of Physiology and Neuroscience, Institute of Functional Genomics, INSERM U1191, CNRS UMR5203, University of Montpellier, 34094 Montpellier, France

**Keywords:** glucocorticoids, glucocorticoid receptor, glucocorticoid receptor ligands, glucocorticoid receptor phosphorylation, glucocorticoid receptors in the brain

## Abstract

Glucocorticoids via the glucocorticoid receptor (GR) have effects on a variety of cell types, eliciting important physiological responses via changes in gene expression and signaling. Although decades of research have illuminated the mechanism of how this important steroid receptor controls gene expression using *in vitro* and cell culture–based approaches, how GR responds to changes in external signals *in vivo* under normal and pathological conditions remains elusive. The goal of this review is to highlight recent work on GR action in fat cells and liver to affect metabolism *in vivo* and the role GR ligands and receptor phosphorylation play in calibrating signaling outputs by GR in the brain in health and disease. We also suggest that both the brain and fat tissue communicate to affect physiology and behavior and that understanding this “brain-fat axis” will enable a more complete understanding of metabolic diseases and inform new ways to target them.

## Introduction

**Figure 1.**
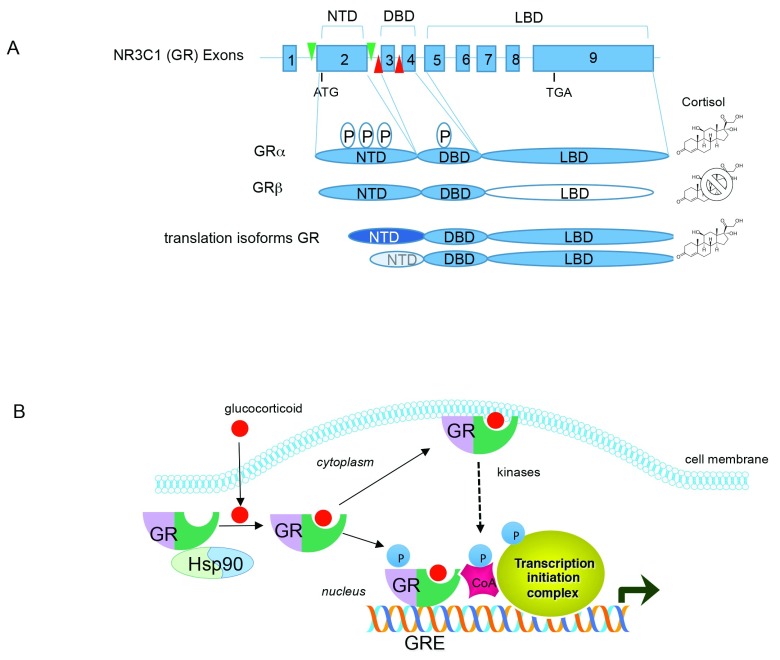
Glucocorticoid receptor (GR) architecture and signaling. ( **A**) Schematic diagram showing the genomic organization of GR gene ( *NR3C1*) and the encoded GR proteins. A “P” within an oval indicates phosphorylation sites (a complete list of GR phosphorylation sites can be found at phosphosite.org). Green and red arrows in the *GR* gene represent LoxP sites engineered into the mouse genome to conditionally delete either exon 2 or exon 3 of *GR*. DBD, DNA binding domain; LBD, ligand binding domain; NTD, N-terminal domain. ( **B**) Signal transduction by GR. Glucocorticoids pass through the cell membrane and bind to the GR/HSP90 complex. Upon ligand binding, HSP90 is released and GR translocates to the nucleus where it can bind DNA and interact with co-activators (CoA) and the transcription initiation machinery to activate gene expression. GR can also repress gene expression by binding to DNA or via protein-protein interactions. For simplicity, only activation by GR is shown. GR can also associate with the cell membrane to evoke rapid signaling via activation of kinase pathways.

The glucocorticoid receptor (GR), a glucocorticoid-dependent transcription factor widely distributed throughout the brain and peripheral tissues, mediates the physiological effects of glucocorticoids. The receptor has a modular architecture characteristic of the steroid receptor family and contains an N-terminal transcriptional activation domain, a central DNA binding domain, and a C-terminal ligand binding domain ( Figure 1A). Upon ligand binding, GR undergoes a conformational change that promotes its release from the heat shock protein 90 complex and translocation to the nucleus, where it modulates the expression of target genes. GR can induce and repress gene expression. The mechanism by which these distinct transcriptional outputs occur likely revolves around allosteric changes in GR evoked by a combination of ligand, DNA binding element sequence, post-translation modifications, and co-activator and co-repressor protein interactions to produce gene-specific activation or repression. These mechanisms have been recently reviewed and will not be re-examined here ^1, 2^.

Adding complexity to GR action is the identification that the GR gene ( *NR3C1*) is alternatively spliced to produce GRβ with an abbreviated ligand binding domain that does not bind any known GR agonists, and acts as a dominant negative inhibitor of GR ^3^. Moreover, alternative translation start sites of the GR mRNA produce a series of N-terminal isoforms in various tissues and are modified post-translationally by phosphorylation to influence gene expression ^4^ ( Figure 1A). Rapid non-genomic actions of GR have also been described in neurons and peripheral tissues ^5^. GR can also modulate mRNA splicing ^6^, mRNA stability ^7^, and microRNA expression and processing ^8^. Thus, GR controls gene expression directly as a transcription factor and indirectly by stimulating signaling pathways that coalesce on GR while also shaping gene expression post-transcriptionally through effects on RNA metabolism ( Figure 1B).

**Figure 2.**
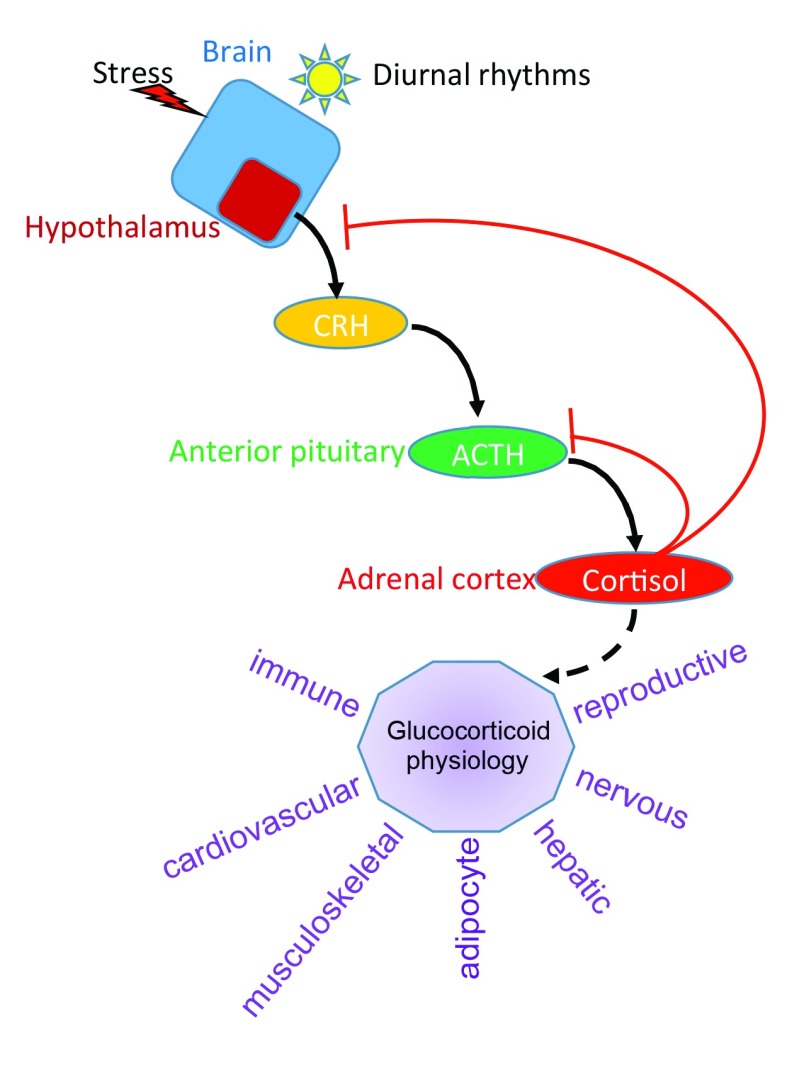
Hypothalamic-pituitary-adrenal (HPA) axis. Shown is a representation of the HPA axis. Light or stress activates the hypothalamus to produce corticotrophin-releasing hormone (CRH). This secreted protein binds to the pituitary gland and induces secretion of adrenocorticotropic hormone (ACTH), which in turn signals the adrenal cortex to produce cortisol. Via a negative feedback loop, cortisol suppresses CRH and ACTH to maintain an optimal, stable level of cortisol in the plasma. In purple are some of the physiological responses affected by glucocorticoids.

The endogenous glucocorticoid in humans is cortisol and is produced by the adrenal gland ( Figure 2). Synthesis of cortisol depends on anterior pituitary-derived adrenocorticotropic hormone (ACTH). Secretion of ACTH is tightly controlled through the hypothalamic-pituitary-adrenal (HPA) axis, where signals (for example, daylight) feed into the hypothalamus to promote release of corticotrophin-releasing hormone (CRH). This in turn promotes ACTH secretion and the synthesis of cortisol from the zona fasciculata of the adrenal gland. As cortisol levels rise, a negative feedback loop is engaged that reduces both CRH and ACTH expression and secretion. This maintains relatively stable levels of plasma glucocorticoids. Cortisol is released in a pulsatile manner throughout the day, and the largest peaks are observed right before awakening (morning for diurnal animals like humans but evening for nocturnal animals like mice and rats), and the secretion of cortisol is linked to circadian rhythm ^9^. Stress, either emotional or physical, results in acute elevated plasma cortisol levels via activation of the HPA axis.

The physiological effects of glucocorticoids and GR are widespread ( Figure 2). GR regulates the nervous, cardiovascular, musculoskeletal, immune, respiratory, reproductive, adipocyte, and hepatic systems, among others. For example, glucocorticoids regulate blood glucose by stimulating hepatic gluconeogenesis, which is how the “glucocorticoid” hormone received its name, via induction of phosphoenolpyruvate carboxykinase ( *PEPCK*) gene ^10^. Glucocorticoids also decrease inflammation and this is due in part to GR’s ability to repress pro-inflammatory gene expression ^11^. This has permitted the use of synthetic glucocorticoids, such as dexamethasone and prednisone, as potent anti-inflammatory drugs. GR suppresses bone formation by a number of mechanisms, including reducing osteoblast differentiation ^12^, inducing osteoblast apoptosis ^13^, and stimulating bone resorbing osteoclasts ^14^, and is a major side effect of pharmacological glucocorticoid administration ^15^. Similarly, adipocytes are sensitive to glucocorticoids. Effects include (1) increased adipogenesis; (2) altered metabolism, including reduced glucose metabolism and decreased lipogenesis under basal or fasted conditions, and increased lipogenesis when glucocorticoids are paired with insulin signaling; and (3) altered adipokine production ^16^. Glucocorticoids in the nervous system are important for physiological homeostasis and response to stress, and an imbalance in GR signaling results in psychiatric disorders (see below).

Given that glucocorticoids are vital for adaptive behaviors upon environmental changes ^17, 18^, GR signaling must be coordinated across tissues and cell types. For example, caloric deficit or surplus and concomitant metabolic adjustments are sensed via glucocorticoid signaling in the liver, pancreas, gut, and adipocytes which culminates in the brain to mediate feeding and satiety ^19^. In the hypothalamus, neurons interact with glia and the vasculature to sense metabolic state ^20^. To control food intake, hypothalamic neurons respond to hormones derived from gut (for example, ghrelin), pancreas (insulin and glucagon), intestine (glucagon-like peptide-1, or GLP-1), adrenal glands (glucocorticoids), and adipocytes (adipokines such as leptin, adiponectin, resistin, and apelin) via cell type–specific receptors ^21^. Disruption of these hormonal signals is a common feature of metabolic disorders and cognitive impairment. Among these, aberrant secretion of glucocorticoids from normally low to chronically high results in metabolic dysregulation featuring fat deposition and impaired synaptic plasticity in neuronal circuits controlling learning and memory. Examples of the pathophysiology of glucocorticoid excess include patients with Cushing syndrome and stress-induced depression and anxiety ^22^.

**Figure 3.**
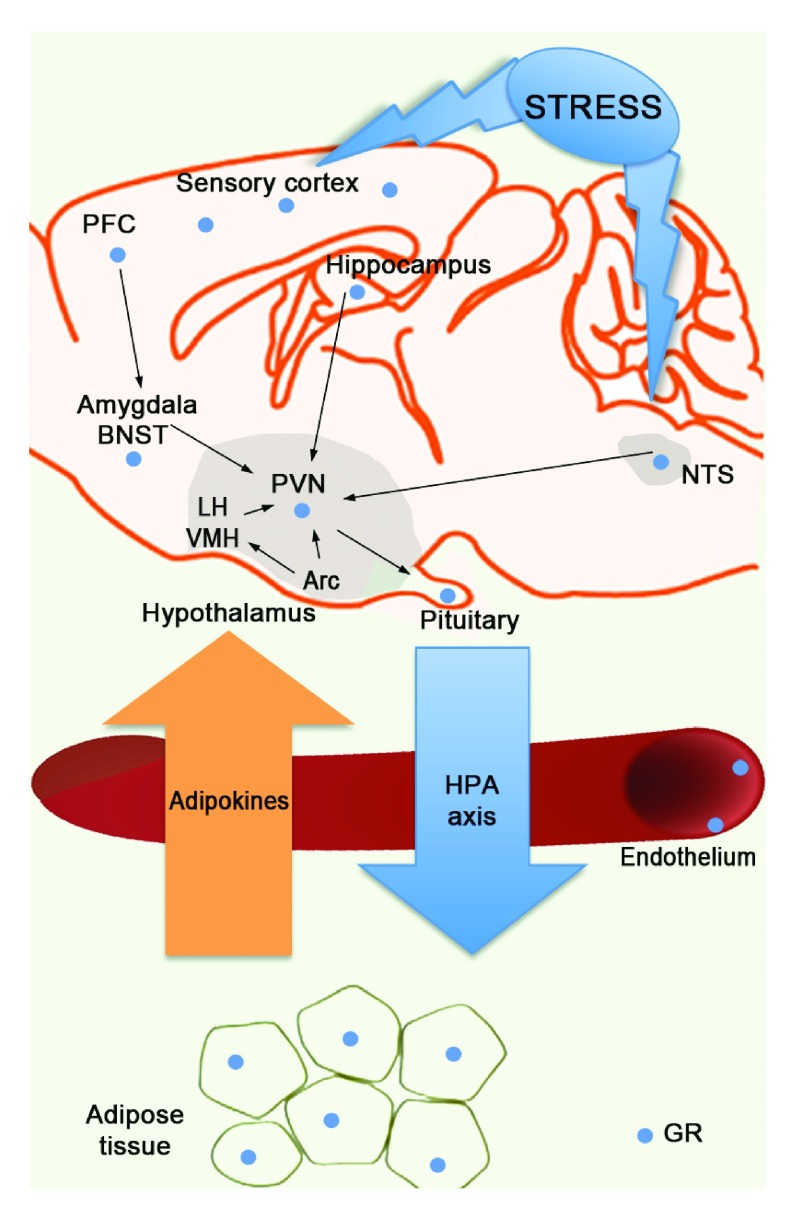
Brain-to-fat signaling axis. Shown is a schematic of the brain and adipose tissue separated by the endothelium. Hypothalamic-pituitary-adrenal (HPA) axis activation promotes glucocorticoid secretion from the brain to the fat. Adipokines secreted from the adipocytes traverse the endothelium and signal to the brain to affect neuronal function. Stress can impact the brain and promote expression of corticotrophin-releasing hormone (CRH) in the paraventricular nucleus (PVN) of the hypothalamus. CRH binds to the pituitary to stimulate the HPA-axis cascade to secrete glucocorticoids to influence the brain and adipocyte function via glucocorticoid receptor (GR) (blue circle). Brain structures that sense stress and signal to the PVN and in turn the pituitary gland are shown and include the sensory cortex, prefrontal cortex (PFC), amygdala/bed nucleus of the strial terminalis (BNST), hippocampus, nucleus tractus solitaries ( *NTS*), arcuate nucleus (ARC), along with the ventromedial hypothalamus (VMH) and lateral hypothalamus (LH).

Recently, our understanding of how GR activity is linked to these important physiological responses has evolved. This is based on new genetically engineered mouse models with alterations in glucocorticoid signaling as well as more sophisticated approaches to physiology and imaging demonstrating glucocorticoids as central effectors of metabolic and neuronal functions. The goal of this article is to review new evidence that GR in adipocytes and in brain contributes to the homeostatic balance of energy metabolism and neuronal plasticity ( Figure 3). This is accomplished by communication between adipose tissue and the brain via adipokines and from the brain to adipocytes by glucocorticoids via activation of the HPA axis. Environmental challenges from caloric excess or chronic stress (or both) can disrupt this axis and can affect tissue sensitivity to glucocorticoids, leading to aberrant GR signaling in target tissue with pathological consequences.

## Loss of function of glucocorticoid receptor in the brain and fat and its impact on physiology

Targeted deletion of *GR* in various cell types of the brain as well as in adipocytes has illuminated our understanding of the impact of GR on adaptive physiology and behavior ^23– 25^. A list of targeted *GR* deletions in the brain, adipocytes, and liver and their resulting phenotypes on the HPA axis, response to high-fat diet feeding, metabolic syndrome as well as effects on anxiety and depression are shown in Table 1.

**Table 1.** Summary of the glucocorticoid receptor tissue-specific null mice discussed in this review.

Study author	GR deletion	GR flox	CRE driver	Phenotype					Reference
				HPA axis	High-fat diet	Dexamethasone treatment	Anxiety	Depression	
De Kloet *et al*.	Adipocyte	Exon 2	Adiponectin	High, resistant to DST	Protected	ND	ND	ND	40
Mueller *et al*.	Adipocyte	Exon 3	Adiponectin	ND	Protected	ND	ND	ND	42
Bose *et al*.	Adipocyte	Exon 3	Adiponectin	No change	Not protected	Mild protection	ND	ND	44
Bose *et al*.	Liver	Exon 3	Albumin	No change	Not protected	Moderate protection	ND	ND	44
Desarzens and Faresee	Adipocyte	Exon 3	Adiponectin	ND	Not protected	Not protected	ND	ND	45
Hartmann *et al*.	Glu neurons	Exon 3	CamK2a	High	ND	ND	Increased	No change	28
Hartmann *et al*.	Glu Neurons	Exon 3	Nex	High	ND	ND	Increased	No change	28
Hartmann *et al*.	GABA neurons	Exon 3	DL5/6	No change	ND	ND	No change	No change	28
Hartmann *et al*.	Amygdala	Exon 3	AAV- CamK2a-cre injection	No change	ND	ND	Reduced fear	No change	28
Kolber *et al*.	Amygdala	Exon 2	AAV-cre injection	No change	ND	ND	Reduced fear	No change	29
Schmidt *et al*.	Pituitary	Exon 3	POMC	No change in adult	ND	ND	No change	No change	32
Wagner *et al*.	Pituitary	Exon 3	POMC	High in juveniles	ND	ND	No change	No change	33
Jeanneteau *et al*.	PVN	Exon 2	Sim1	High	ND	ND	ND	ND	30
Laryea *et al*.	PVN	Exon 2, exon 3	Sim1	High	ND	ND	No change	Increased despair	31

CRE, Cre recombinase; DST, dexamethasone suppression test; GR, glucocorticoid receptor; HPA, hypothalamic-pituitary-adrenal; ND, not determined; PVN, paraventricular nucleus.

### Glucocorticoid receptor inactivation in the brain

Deletion of *GR* in the forebrain (Camk2a-Cre), a region encompassing the cerebral hemispheres and hippocampus that control many of our senses, resulted in HPA-axis hyperactivity and impaired negative feedback regulation and increased depression and anxiety ^26, 27^. This is direct evidence that GR in the forebrain participates in the HPA-axis activity to control depression and anxiety. By contrast, deletion of *GR* from the central nucleus of the amygdala (Cre expressing virus injected into *GR* floxed mice), a structure within the limbic system that is responsible for emotions (including fear), had no effect on the HPA axis but reduced fear ^28, 29^. Although a deletion of *GR* in the paraventricular nucleus (PVN) of the hypothalamus (Sim1-Cre) had no effect on anxiety or cognition, it did result in the dysregulation of the HPA axis ^30, 31^. Similarly, the inactivation of *GR* gene ** in the pituitary gland (POMC-Cre) resulted in aberrant HPA-axis activity but without affecting anxiety or cognition ^32, 33^. Combined inactivation of *GR* in both hypothalamus and pituitary results in extreme dysregulation of the HPA axis and is not consistent with life ^34^.

To test the relevance of GR to specific neuronal circuits, *GR* was selectively inactivated in dopaminoceptive neurons (Drd1-cre). This resulted in social aversion and reduced drug-seeking behavior without affecting anxiety or HPA-axis activity ^35– 37^. Inactivation of *GR* in glutamatergic neurons of the forebrain (Nex-Cre) deregulated the HPA axis and reduced fear, whereas deletion of *GR* in GABAergic neurons (Dl5/6-Cre) affected neither the HPA axis nor response to fear ^28^. Taken together, these results suggest that the actions of GR on the regulation of the HPA axis and synaptic physiology, circuitry, and behavior are cell type–dependent ^38^. To produce these effects, GR deploys both rapid non-genomic mechanisms affecting neurotransmitter signaling and slower genomic actions that alter transcription to provide morphological (synapse and cytoskeleton) and metabolic (mitochondria) adaptation ^39^. In addition, GR responds to signals from the environment by post-translationally modifying the receptor (for example, by phosphorylation) and this conveys contextual differences with the potential to alter GR transcriptional programs and ultimately physiology and behavior.

### Glucocorticoid receptor inactivation in adipocytes

Multiple adipocyte-specific *GR* knockout mice have been generated and assessed for effects on the HPA axis and metabolism, including protection against diet-, age-, or dexamethasone-induced obesity ( Table 1). The first fat-specific *GR* knockout mouse (adiponectin-Cre) was reported by de Kloet *et al*. ^40^. They observed changes in the regulation of the HPA axis, including increased secretion of glucocorticoids following acute stress and decreased response to exogenous glucocorticoid suppression, suggesting a role for adipocytes in the negative feedback of the HPA axis. Theoretically, this could happen via a loop involving sensory innervation of adipose tissue ^41^ or, as the authors suggested, occur as a result of “leaky” expression of the Cre recombinase in a non-adipocyte cell type. In addition, these mice were protected from diet-induced obesity. This suggests communication between the fat cells and the brain in regulating metabolism and the HPA axis.

Mueller *et al*. also developed an adipocyte-specific *GR* knockout mouse (adiponectin-Cre) and too found that diet- and age-associated obesity was reduced ^42^. The impact on the HPA axis was not examined. They also performed metabolomics from serum and found differences in metabolite abundance in fed and fasted states between wild-type and adipocyte-specific *GR* knockout mice. For example, under steady-state conditions, the abundance of certain fatty acid species and branched-chain amino acids was increased in the fat-specific GR knockout mouse. Another interesting phenotype displayed by this *GR* knockout mouse was resistance to lipolysis during fasting such that adipose depot mass was preserved at the expense of lean mass in the knockout compared with wild-type mice. The lipolytic defect was studied further *in vitro* and was seen with adrenergic agonists but not direct activators of adenylate cyclase. This led the authors to suggest that there was an alteration in the signaling between the adrenergic receptor and adenylate cyclase, specifically at the level of G-proteins. This might suggest a level of crosstalk between GR and G-proteins in fat cells. Such a link between arrestins, well-known regulators of G-protein signaling, and GR has been shown previously ^43^.

In addition, in the adipose-deficient *GR* knockout mice, the authors observed reduced liver steatosis, protection against pyruvate overload, and increased insulin sensitivity. This is likely due to changes in lipolysis and reduced fatty acid trafficking to the liver and might explain in part the reduced fat mass on high-fat diet. These findings also imply a link between adipocytes and liver that is mediated by GR.

To directly compare the contribution of adipocyte versus ** hepatic *GR* inactivation to diet-induced obesity and glucocorticoid-mediated metabolic syndrome, Bose *et al*. created both fat-specific and liver-specific *GR* knockout mice ^44^. Whereas the fat-specific *GR* knockout mice (adiponectin-Cre) were only mildly protected from metabolic dysfunction induced by high-fat diet or dexamethasone, the protection was more evident in liver-specific *GR* knockout mice (albumin-Cre). This suggests that liver GR is also an important conduit in the development of metabolic syndrome elicited by caloric excess and dexamethasone. Bose *et al*. also uncovered a homeostatic mechanism to compensate for the loss of GR in the liver such that the kidney increased the expression of gluconeogenic enzymes when treated with dexamethasone, an effect not seen in wild-type mice. This reveals an unexpected mechanism that compensates for the loss of GR activity in one tissue by another to maintain metabolic integrity.

A recent study by Desarzens and Faresee demonstrated that when *GR* was deleted in adipocytes (adiponectin-Cre), there was little effect on body weight or adipose tissue growth when challenged with a high-fat, high-sucrose diet ^45^. However, *GR* inactivation in adipocytes upon high-fat, high-sucrose diet did result in enhanced macrophage infiltration, increased inflammation, and modified glucose tolerance. This shows that in addition to the cell-autonomous effect of GR on adipocytes, there are cell–non-autonomous effects of GR on adipocyte biology via modulation of the inflammatory response.

It is well recognized that sequence-specific transcription factors, such as GR, interact with other transcription factors to control gene expression under particular metabolic or environmental states ^46, 47^. In fact, it was recently shown that the forkhead box protein A3 ( *FOXA3*) not only is regulated by GR in adipose tissues but is required for the binding of GR to a subset of its target genes to promote the physiological response of glucocorticoids in adipocytes ^48^. Functionally, removing *FOXA3* from the fat protected against dexamethasone-induced obesity without affecting the pathological response of chronic glucocorticoid treatment in other tissues. This indicates that GR and FOXA3 cooperate to promote fat expansion upon chronic dexamethasone treatment.

Although there are similarities among the studies with respect to metabolic phenotypes of *GR* adipocyte-inactivation, differences were also observed. This likely reflects variations in the *GR* floxed alleles employed, genetic backgrounds, diets, age of the mice, or additional uncontrolled factors such as the microbiota. For example, the study by de Kloet *et al*. used *GR* exon 2 floxed mice which may not promote full recombination with some Cre lines ^31^. Moreover, *GR* exon 2 “deleted” mice have been shown to produce residual GR protein in the form of a truncated GR that lacks the N-terminal activation domain (this domain is contained within exon 2). This portion of GR still contains the DNA and ligand binding domains and remains competent for signaling and modulating the expression of a subset of genes ^49^. It is not clear from Bose *et al*. why their adipocyte-specific *GR* knockout mice were only mildly protected from diet-induced obesity. Likewise, the study by Desarzens and Faresee failed to demonstrate any protection against diet-induced obesity of *GR* inactivation in adipocytes. One possibility is the difference in age at which the mice were placed on a high-fat diet. In addition, all of these studies used different high-fat diets. Another potential confounder is the difference in gut microbiota between mice housed at different institutions, which could affect the outcome ^50^. This reflects the complexities of designing *in vivo* experiments to determine the impact of *GR* deletion in adipocytes to physiological responses to diet.

## Glucocorticoid receptor insufficiency (glucocorticoid resistance) in the brain-fat axis

Decreased tissue responsiveness to glucocorticoid is common in human diseases (inflammatory, immune, neuropsychiatric, and neurodegenerative) characterized by a state of excessive secretion of glucocorticoids due to the loss of feedback inhibition of the HPA axis by defects in GR signaling ^51, 52^. This state of glucocorticoid resistance, which can result from chronic stress ^53^, coincides with decreased expression of brain-derived neurotrophic factor (BDNF) in the cortex and hippocampus and increased expression of BDNF in amygdala and dopaminergic neurons ^54^.

Phosphorylation of GR has been suggested to be a mechanism contributing to glucocorticoid resistance in multiple disease models ^55^. Although a majority of GR phosphorylation is glucocorticoid-dependent ^56^, recent data indicate that GR phosphorylation can also be glucocorticoid-independent. This implies that GR activity could be influenced by signals in addition to glucocorticoids ^57, 58^. For example, the activation of the BDNF-TrkB pathway results in phosphorylation of the human GR at serine 134 (S134) (conserved in rat GR S155 and mouse GR S152), thereby fostering the recruitment of co-factor proteins (for example, CREB1) and changing the target genes in response to glucocorticoid stimulation ^57, 58^. TrkB is a receptor tyrosine kinase that upon binding of neurotrophins, such as BDNF, elicits downstream signaling events, including activation of the mitogen-activated protein kinases, to affect the connectivity of neuronal circuits. This molecular pathway, among others triggering GR phosphorylation at S134, could provide a cell- and signal-dependent context to GR signaling. In fact, we have shown that the crosstalk between BDNF-TrkB signaling and the glucocorticoid-GR pathway in neurons alters the repertoire of genes transcribed by GR through changes in GR phosphorylation ^58^. This suggests that disruption of BDNF expression in the brain would compromise GR signaling. Consistent with this idea, chronic stress, which decreases BDNF levels in cortex, decreased GR phosphorylation at S134 with effects on synapse number in cortex ^59^. Furthermore, deletion of a BDNF-sensitive GR phosphorylation site in cortical neurons resulted in glucocorticoid resistance in mice and reduced expression of GR target genes (for example, DUSP1), decreased numbers of synapses, and promoted Tau phosphorylation ^60^. Such crosstalk appears to be physiologically relevant in humans as DUSP1 expression and markers of synapses in the cortex correlated with cognitive performance in human subjects with diagnosed cognitive impairment.

It is noteworthy that the BDNF-dependent GR phosphorylation sites reside near a caspase 1 (CASP1) cleavage site in GR that is responsible for glucocorticoid resistance observed in acute lymphoblastic leukemia ^61^. Mechanistically, by virtue of lower methylation of the CASP1 promoter in glucocorticoid-resistant leukemic cells, CASP1 expression becomes elevated, which in turn mediates the cleavage of GR at its N-terminal transactivation domain causing partial loss of GR transcriptional activity. This is sufficient to produce glucocorticoid resistance. Consistent with this idea is the finding of a GR variant lacking the N-terminus in the selection of mouse lymphoma cells resistant to glucocorticoid-induced cell death ^62^. CASP1 inhibitors are promising compounds to explore for alleviating glucocorticoid resistance disorders with a chronic inflammatory component. Whether this holds in models of neuropathology or metabolic disease has not been explored.

Glucocorticoid resistance by alterations in GR activity as a function of stress and linked to changes in GR phosphorylation has also been observed *in vivo*. For example, a recent analysis of GR-mediated transcriptional activity through a GRE-linked luciferase reporter gene in the hippocampus of mice revealed decreased GR activity upon exposure to chronic stress despite high levels of circulating glucocorticoid ^63^. Remarkably, treatment with the anti-depressant fluoxetine restored GR transcriptional activity and corrected behavioral deficits induced by chronic stress. Phosphorylation of the N-terminal transcriptional activation domain of GR correlated with treatment efficacy ^59^. These results indicate that in stress-induced depressive-like disorder, defects in GR signaling operate at least in part through changes in receptor phosphorylation. Therefore, therapeutic strategies aiming at enhancing GR signaling directly with selective agonists or indirectly via conditioning pathways (for example, BDNF) are promising options. Whether changes in glucocorticoid sensitivity by affecting GR phosphorylation through extracellular signals are evident in adipocytes to influence metabolic responses has not been explored. It is tempting to speculate that the differential response in adipocytes to glucocorticoids in the absence and presence of insulin could reflect alterations in GR phosphorylation and transcription reprograming via crosstalk with the insulin signaling pathway akin to what we observed for BDNF-mediated signaling effects on the GR response in neurons ^58^.

Chronic stress, which activates the HPA axis to raise glucocorticoid levels systemically, promotes both psychiatric and metabolic disorders as a result of impaired synaptic plasticity of brain circuits that mediate reward ^36, 64^. In fact, reinforcement by stress of the reward circuitry enhances the consumption of highly caloric foods ^65^. Conversely, limiting the consumption of food can reverse HPA-axis dysregulation and improve behavior upon stress ^19^. Consistent with the reciprocal effects of the BDNF-TrkB and glucocorticoid-GR pathways on the regulation of the HPA axis ^54^ is that deletion of the BDNF receptor TrkB in cholecystokinin (CCK)-GABAergic neurons resulted in HPA-axis hyperactivity and obesity in mice ^66^. Although it is possible that some of the CCK neurons synapse directly with the PVN, it is more likely that the effect of these neurons is indirect and relayed through other structures that signal to the hypothalamus to affect HPA-axis activity. Importantly, blocking BDNF signaling in CCK neurons induced glucocorticoid resistance, resulting in increased CRH expression, elevated plasma glucocorticoid levels, adrenocortical hyperplasia, glucose intolerance, and enhanced lipogenesis reminiscent of patients with Cushing syndrome ^66^. In the hypothalamus, CRH expression is induced by activation of the CREB transcription factor through BDNF-TrkB signaling via activation of protein kinase A. CRH expression is repressed by glucocorticoids due in part to GR binding to CREB and repressing its transcriptional activity and through GR interfering with the nuclear import of the CREB co-activator CRTC2 ^30, 58^. Behaviorally, a functional interaction between the BDNF-TrkB and glucocorticoid-GR pathways has been demonstrated to be essential to learn inhibitory avoidance, contextual fear, coping with stress, and control of the appetite balance ^67– 69^.

## Moving forward: linking glucocorticoid receptor-regulated brain function and metabolism

The contributions of GR in the brain and peripheral tissues involved in metabolism are not mutually exclusive, and many studies highlighted here have begun to shed light on these actions. The next frontier of GR research *in vivo* will continue to meld these areas. A polymorphism in BDNF (Val66Met) that diminishes BDNF secretion and signaling results in enhanced anxiety ^70^ and alterations in vulnerability to stress ^71^ and energy balance and obesity in humans and rodents ^72^. Therefore, it would seem important to examine the effects of BDNF/GR crosstalk by testing how mice with the BDNF Val66Met allele compare with mice lacking the BDNF-sensitive GR phosphorylation sites in various regions of the brain to protect or exacerbate anxiety- or diet-induced obesity or both. In addition, behavioral response to food preferences and satiety could be assessed in such models. Conversely, alterations in glucocorticoid signaling pathways in metabolic tissues could be modeled in combination with alterations in neurotropic pathways to determine the crosstalk between GR activity in the brain and metabolic tissues. In fact, BDNF is present not only in brain but also in blood at high levels, indicating possible peripheral effects of BDNF signaling on GR ^73^. Therefore, desynchronization of BDNF/GR axis could impact multiple physiological functions (for example, inflammatory, immune, metabolic, and cognitive) and escalate vulnerability to stress-induced illnesses. Although these pathways are often investigated separately, future research will need to consider GR signaling in an integrated manner to better understand homeostatic and pathological processes modulating GR action and to harness this information for therapeutic benefit.
